# Job attributes affect the relationship between perceived overqualification and retention

**DOI:** 10.1186/s43093-022-00147-3

**Published:** 2022-09-16

**Authors:** Maria Piotrowska

**Affiliations:** Wroclaw University of Economics and Business, ul. Komandorska 118-120, 53-345 Wroclaw, Poland

**Keywords:** Overqualification, Organizational commitment, Retention, Nonwage benefits, M54, J63

## Abstract

The paper explores the possibility of reducing the effect of perceived overqualification on employee retention (i.e., turnover intentions and job search behaviors) through non-salary (nonwage benefits, elasticity of work hours, and procedural justice) and salary (pay satisfaction) attributes of work. The problem of overqualification arises when the skills and experience or the knowledge and education of an employee are higher than those required for the job that the employee performs. This situation may induce an employee to leave the organization. This research uses the concept of perceived overqualification and addresses three unresolved issues regarding salary and non-salary job attributes which can modify the effects of perceived overqualification. These issues include the mechanism through which the aforementioned effects are transferred onto retention, interpersonal justice as a moderator, and the importance of negative affectivity, which may be responsible for the relationship between perceived overqualification and employee retention. The study uses conditional analysis of the process developed by Hayes and data from a survey conducted among 100 overqualified employees, who were identified among 826 randomly selected people in Poland. The findings show that non-salary job attributes can be a better instrument than salary in increasing the organizational commitment perceived by overqualified employees. A stronger organizational commitment prevents the overqualified from leaving the organization. As regards the relationship between perceived overqualification and turnover intention, the moderation impact of interpersonal justice (respect from supervisors) is stronger than that of pay satisfaction. Negative affectivity does not create the common tendency in perceived overqualification and retention. Based on the findings, I propose several practical recommendations.

## Introduction

Overqualification means that an employee has more skills, expertise, abilities, education, experience, and other qualifications than are needed for the job [27, p. 217]. The international workforce is increasingly educated and skilled, e.g., 40.7% of the 30–34 year-old adults in the EU attained tertiary education in 2018, hitting one of the Europe 2020 targets for the first time (source: Eurostat (data code online: t2020_41). Thus, the target that 40% of the EU population aged 30–34 should obtain tertiary education by 2020 was achieved two years ahead of schedule.

There has been a disturbing tendency in the labor market to widen the scissors of supply and demand for job applicants with higher education diplomas. The growing number of people with higher education is accompanied by a decreasing demand for highly qualified employees, e.g., Vedder et al. [104] show that almost half of graduates of US colleges work in positions that do not require higher education. The decreasing demand for employees with higher education results from the cost minimization strategy used by and increasing number of companies. This leads to employees with higher education in positions that do not require such high qualifications. As a consequence, more and more people in the labor market are becoming overqualified. This trend may be exacerbated by the covid-19 crisis. Some of the companies that will experience financial problems may choose the strategy of minimizing costs. They will be faced with the problem of how to prevent overqualified employees from leaving their organization without offering higher wages. Which non-salary attributes of work (nonwage benefits, elasticity of work hours, and procedural justice) can convince overqualified employees to stay in their companies and continue working in positions below their qualifications. Therefore, insight is needed into how non-salary attributes of work affect perceived overqualification (POQ).

Much of the literature on management psychology and occupational psychology focuses on perceived overqualification (POQ) rather than on objective overqualification. Perceived overqualification (POQ) is related to the perceptions of employees that they are better educated and have more experience and/or knowledge, skills and abilities (KSA) than needed for their position [26, 47], whereas objective overqualification means that the level of education and skills of the employee is higher than required by the standards describing the job in question. [32, 70].

POQ is measured through employee surveys. Objective overqualification is expressed by comparing the level of skills and preparation of an employee with the level of requirements for a given occupation. Both levels are assessed by external persons using appropriate measurement scales. In their meta-analysis of perceived overqualification, Harari et al. [39] provide two arguments showing that perceived overqualification (POQ) reflects the essence of overqualification better than objective overqualification (see also [72].

First, POQ expresses the personal experiences of overqualified employees much better than objective measures [27]. Second, the indicators used to assess objective overqualification fail to capture the differences between positions with the same name [70]. Taking both arguments, my research focuses on the subjective measure of overqualification, or perceived overqualification (POQ).

In their meta-analysis, Harari et al. [39] show that in the literature to date, POQ was mainly presented in terms of identifying factors and explaining the effects (see references in the cited meta-analysis). There are still issues that should be further explored. Harari et al. [39] indicate three such areas of research. The first concerns the direct and indirect effects of POQ on turnover intentions, the second relates to the impact of negative affectivity on POQ and the assessment of job attributes, and the third focuses on the identification of moderators important for POQ outcomes.

This study refers to these three unresolved issues in context of salary and non-salary job attributes which could modify the effects of POQ. Overqualified employees, as all employees, care about their salaries but also about nonwage benefits and flexible working hours, and they are also sensitive to fairness at their workplaces. These non-salary job attributes can matter when such overqualified employees decide to leave their organizations. In general, this study should answer the question of whether it is possible to reduce the impact of POQ on employee retention through salary and non-salary job attributes. Thus, there are three objectives of this research paper.

The first objective is to investigate the mechanisms through which POQ affects retention, including interpersonal justice as a moderator. The second objective is to compare the mediation effects of salary and non-salary job attributes (nonwage benefits, flexible work hours, and procedural justice) in the relationship between POQ and the employee’s attachment to his or her organization. The third objective is to examine the impact of negative affectivity (pessimism) on POQ and perceptions of job characteristics. By investigating the significance of negative affectivity (NA), as a correlate of POQ, it is possible to assess whether the obtained relationships are spurious or not. It may be the case that NA is the third variable that produces POQ, organizational commitment, and perceptions of job attributes.

The micro-level data needed to conduct this research was collected as part of a questionnaire survey carried out on 826 Poles aged 25–45, i.e., in mobile working age, who had obtained a master's or bachelor's degree.

Overqualified employees have been identified from among all participants of the study on the basis of their subjective assessment of how their current job matches their skills and experience (or knowledge and education). The number of the overqualified participants is 100 (90).

The research approach uses the conditional process analysis developed by Hayes [41], which includes mediation analysis and moderation analysis. The former is used to estimate the model of the mechanism through which the effect of the independent variable is transmitted to the dependent variable. The latter helps to identify factors that may strengthen or weaken this transmission mechanism.

The findings of this research broaden our knowledge of perceived overqualification (POQ) by shedding light on unclear issues such as mediation effects of POQ and the fear that the relationship between POQ and retention is spurious, i.e., it follows that both POQ and retention are correlated with the "third" variable, which is the negative affectivity. The findings also broaden the knowledge about POQ effects, showing the role of non-salary job attributes as tools that can prevent overqualified employees from leaving their organization as well the relevance of interpersonal justice as a moderator. Finally the research reveals the mechanisms through which POQ influences job search behaviors.

The paper consists of ten sections. "Theoretical framework for the relationship between overqualification and turnover intention" section presents two main theories which are a base for the relationship between overqualification and turnover intention. "Hypothesis development" section presents the literature in which hypotheses are embedded. Section four describes the materials and the method. "Results" and "Discussion" sections present and discuss the results, Sect. "Research contribution" section sums up research contribution. "Limitations and further studies" section discusses limitations of the research and explores further avenues of study. "Recommendation for practice" section offers practical recommendations. Finally conclusions summarize the findings.

## Theoretical framework for the relationship between overqualification and turnover intention

Research into the negative effects of overqualification is based mainly on two theories—the relative deprivation theory [22] and the person-job fit theory [52]. Relative deprivation theory is the basis for explaining the negative influence of overqualification on employees and consequently the negative effects for organizations [27], while Person-Job (P-J) fit theory helps to understand the effects of overqualification on job attitudes [62].

Employees whose qualifications and professional experience are not used at work have a sense of relative deprivation. Their work-related frustration stems from a comparison of their own working conditions with those of their peers. This is because they expect a similar social status, pay and benefits, and interpersonal relationships [103] but they feel their jobs do not satisfy these expectations. This discrepancy between expectations and reality generates anger and resentment. Relative deprivation theory is useful in explaining the emotional reactions of employees to perceived overqualification, for example, Erdogan et al. [28] found that relative deprivation is a mediator in the relationship between perceived overqualification and subjective well-being.

The Person-Job (P-J) fit theory, as a theoretical framework in studies on the effects of overqualification on job attitudes [62], helps to understand the links between overqualification and turnover intentions [72]. The Person-Job (P-J) fit theory is part of a broader approach to matching the employee with the workplace, which is the person-environment (P-E) fit theory [52]. The P-E fit theory concentrates on the fit, or the match, between characteristics of an employee and the characteristics of his or her job. A good fit generates positive outcomes for the employee and the organization such as higher job satisfaction and stronger organizational commitment, better job performance and career success [52, 83], while a poor fit creates undesirable outcomes, such as turnover intention [53,94]. Person-Job fit, one of the forms of P-E fit, refers to the compatibility between knowledge, skills, abilities, and other characteristics (KSAOs) of the employee and the demands and characteristics of the job. Researchers found a robust and negative relationship between P-J fit and both turnover intentions and job search behavior. With respect to turnover intention, this negative link was confirmed by Bretz and Judge [15], O’Reilly et al. [82]. In their meta-analysis, Kristof-Brown et al. [54] also indicate a robust negative link between P-J fit and the intent to leave the organization (ρ = − 0.46). The negative effects of P-F fit on job search were confirmed by [17, 18] as well as Schneider et al. [96].

The relevant literature distinguishes two types of P-J fit: demands-abilities fit and needs-supplies fit Cable and DeRue [23]. The former pertains to the match between the requirements of the job and the KSAOs of the employee whereas the latter, to the degree to which the needs and expectations of an employee are satisfied by the job offered by the organization. According to the definition, overqualification is a particular case of poor demands-abilities fit. The overqualified employees have excessive education, experience and/or skills relative to job demands. The result of such a poor fit of KSAOs experienced by an overqualified employee are negative job attitudes (job dissatisfaction and weak organizational commitment) and retention (turnover intentions and job search behaviors).

The P-J fit literature indicates the mediating role of the needs-supplies fit [66]. A mismatch between what the job provides and what an employee needs from his or her job can be a mediator in the relationship between overqualification and its negative effects.

In his review of the literature, Zhang [112] points to other theories related to overqualification: Organizational Justice theory, Conservation of Resources theory, Effort-Reward Imbalance theory. These will be used to justify the moderators used in the current study.

## Hypothesis development

This research explores two concepts related to employee retention. The first concerns turnover intention, which is the employee's willingness to leave the organization. The second concept pertains to specific actions aimed at finding a new job. Two types of such job search behaviors are considered in the study: browsing job offers in the press and on the Internet, and applying for a new job.

In the first stage of the study, I try to answer the question of how overqualification leads to retention. Based on previous research on overqualification and with the application of P-J fit theory and Organizational justice theory as a theoretical framework, I suggest, with regard to the relationship between perceived overqualification and turnover intention, that (1) perceived overqualification will be positively associated with turnover intention, (2) this relationship will be entirely mediated through organizational commitment, (3) this indirect effect will be moderated by interpersonal justice and pay satisfaction. With respect to the relationship between perceived overqualification and job search behavior, I argue, that (1) perceived overqualification will be positively associated with the browsing of job offers in the press and on the Internet, (2) this relationship will be entirely mediated through organizational commitment, (3) perceived overqualification will directly affect applying for a new job, 4) this direct effect will be moderated by interpersonal justice and pay satisfaction. Given the significant role of organizational commitment, as a mediator, in the second stage of the study I test whether using different non-salary job attributes it is possible to reinforce organizational commitment and thus mitigate the negative effects of overqualification on retention. Finally, to validate if the obtained results are robust, I test whether the negative affectivity (pessimism) has a significant impact on both the perception of overqualification and on the overqualification effects.

The starting point for this study is the relationship between overqualification and retention. Literature on overqualification provides a number of consistent results indicating a positive relationship between perceived overqualification and turnover intentions. Among the researchers who confirm that overqualification contributes to a stronger intention to leave the organization are Burris [16], Bolino and Feldman [13], Wald [105], Maynard et al. [74], McGuinness and Wooden [76]. A number of empirical studies also confirmed that overqualification was associated with active job search behavior [30, 31, 72, 105].

Although there is robust empirical evidence on the how overqualification and retention are related and all studies show a negative link, there are no unequivocal results in the literature that would confirm the nature of this relationship, i.e., that POQ directly affects turnover intention or that the POQ effect is indirect through a mediator. This raises the question of what kind of mediator could transmit the effect of overqualification on retention. According to the meta-analysis by Harari et al. [39], the POQ affects turnover intention both directly and through organizational commitment. In classic models of turnover [71], organizational commitment is the key factor affecting the withdrawal process. These models also often indicate that low commitment stems from job characteristics such as low opportunity for promotion, low autonomy and a high degree of routinization, which ultimately end in the employee searching for a new job [86–88]. Precisely these job characteristics are associated with overqualification. A dissatisfaction with one’s current job and, consequently, with the organization in which an employee works, prompts him or her to look for a more suitable job. A poor rating of an organization leads to a low organizational commitment. This happens very often to overqualified people. Organizational commitment is also an important mediator in more recent models of turnover [58, 68]. Ahuja et al. [3] even shows that organizational commitment is the strongest predictor of employee turnover intention. Based on the findings in the turnover literature, this research concentrates on organizational commitment, and more precisely on affective commitment as a mediator of the relationship between perceived overqualification and retention.

Organizational commitment is a bond that develops between an employee and his or her organization. High organizational commitment encourages the employee to engage more deeply in the work, makes him or her loyal to the employer and makes the employee share the values that guide the organization [93]. Research on organizational commitment often focuses on its three dimensions: the employee’s emotional attachment to his or her organization (the affective dimension), the perceived costs of quitting (the continuance dimension), and one’s feeling the he or she has an obligation to stay with the organization (the normative dimension for details see Allen and Meyer, [4]. A review of the theoretical and empirical literature on organizational commitment convinced Mercurio [77] to conclude that emotional, or affective commitment is the core essence of organizational commitment.

These three dimensions of organizational commitment tell us that employees stay with the organization because they want to, need to, or ought to. This research concentrates only on the affective organizational commitment (AOC) that occurs when an employee wishes to continue working in an organization, identifies with it, and desires to remain a part of it. The employee becomes committed to an organization because he or she "wants to." This focus on the affective organizational commitment is a consequence of the second research objective, i.e., the comparison between the mediation effects of salary and non-salary job attributes (nonwage benefits, flexible working hours, and procedural justice) in the relationship between POQ and the employee’s attachment to his or her organization. The results of the study are expected to answer the question: If offered non-salary job attributes, will overqualified employees want to stay with the organization?

The affective organizational commitment (AOC) plays an important role in explaining the impact of perceived overqualification on retention because AOC, on the one hand, is a crucial outcome of perceived overqualification and on the other, is an essential factor that affects turnover intention and job search behavior. Based on their meta-analysis of perceived overqualification Harari et al. [39] indicate that AOC is one of the most studied outcomes of perceived overqualification and, what is important, these studies show relatively consistent results. Overqualification is related to low affective organizational commitment. Feldman et al. [30] and Maynard et al. [74], and Maynard and Joseph [73] demonstrate the negative correlation between POQ and AOC. A review of empirical studies also confirms a significant relationship between AOC and retention. The findings of an analysis of multiple studies conducted by Cooper-Hakin and Viswesvaran [21] revealed that affective organizational commitment is negatively correlated with turnover (ρ = − 0.20). Lin and Chen [60] and Wu and Polsaram [107] as well as Cave et al. [19] also show a negative relationship between AOC and employee’s turnover intentions. Somers [99] indicates AOC as a predictor of job search behavior.

When examining affective organizational commitment as a mediator in the relationship between perceived overqualification and turnover intention it is important to determine whether POQ affects turnover intention entirely or partly through organizational commitment. If the effect of POQ was entirely mediated by organizational commitment, then its reinforcement through job attributes would more effectively prevent overqualified employees from leaving the organization.

Thus, I hypothesize that the mechanism through which POQ affects turnover intention is as follows:

### **H1**

The relationship between perceived overqualification and turnover intention is entirely mediated through organizational commitment.

The first stage of research related to the relationship between POQ and turnover intention will be completed by validating two hypothesis on the moderating roles of interpersonal justice and pay satisfaction. I argue that overqualified employees, despite a weak fit with their job, may still feel they are valuable members of the team if they have a sense of belonging to the organization. Overqualified employees can be expected to be alert to signals about their position in the group they work for. One of the strongest status signals is fairness [102]. Employees who are treated with respect by their superiors have a sense of worth and their co-workers appreciate their position in the team. Deng et al. [24] shows that interpersonal influence is a moderator which makes employees less sensitive to the negative effects of perceived overqualification. In their synthesis of the literature on overqualification, Erdogan and Bauer [25] conclude that because of feelings of deprivation, overqualified employees may isolate themselves from their co-workers. As a result of this distancing, they do not use their higher qualifications or opportunities to help other co-workers. Based on their research on the nature of the relationship between overqualification and social network centrality, Erdogan et al. [29] indicate the possibility to alleviate this tendency. The overqualified employees need to feel that they are part of the team. They must be treated with respect by their superiors. Interpersonal justice, i.e., the respect of their superiors, [11, 38] positively influences relations between employees who feel treated with respect. They identify with their team and they are committed to their organization [5]s. In their review of empirical work on factors of turnover intention, Belete [8] show the results of studies that confirm an inverse and significant relationship between interactional justice and turnover intention [2, 84, 98]. He also cites the publication by Kwai et al. [57], who found that higher organizational justice tends not only to reduce turnover intention but also to increase organizational commitment.

Based on the studies presented in the literature, I argue that interpersonal justice should weaken the effect of POQ on the turnover intention. This will be checked by validating the hypothesis:

### **H2**

The relationship between perceived overqualification and turnover intention is moderated by interpersonal justice.

In order to see which of the two moderators, interpersonal justice or pay satisfaction, has a greater effect on lessening the negative impact of POQ on turnover intention, in addition to hypothesis 2, hypothesis 2a for pay satisfaction will be validated:

### **H2a**

The relationship between perceived overqualification and turnover intention is moderated by pay satisfaction.

The inclusion of pay satisfaction, as a moderator, in this study is dictated by the conclusive results presented in the literature, indicating a significant and negative relationship between pay level and turnover intention (for example [40, 56]). If pay satisfaction proves to be a weaker moderator than interpersonal justice, it will mean that an organization which wants to retain overqualified employees but cannot offer higher salaries, should place special emphasis on treating such employees fairly.

**Fig. 1:**
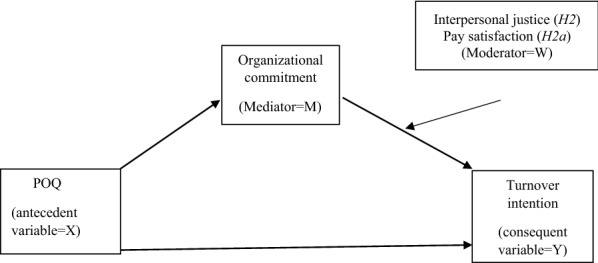
Conceptual model for the mechanism through which POQ affects turnover intention (hypotheses 1, 2, 2a)

Figure 1 presents the mechanism through which POQ affects turnover intention with organizational commitment acting as a mediator and interpersonal justice or pay satisfaction as a moderator.

In developing the first stage of the current study, I focus on the relationship between overqualification and job search behavior. Previous studies related to job search have failed to address the relationship between POQ and behaviors aimed at finding a job[48]. Research has mostly focused on the influence of perceived overqualification on employee turnover [26, 63, 76]. Maynard and Parfyonova [72] expanded this investigation to include the relationship between perceived overqualification and withdrawal with consideration of active job search behavior. Their study was the first to examine affective commitment—as a potential mediator of the relationship between perceived overqualification and job search behavior. They found that affective commitment was a non-significant mediator. I suppose this lack of significance for the overall indirect effect of affective commitment may have been caused by how the job search measure was constructed. Maynard and Parfyonova [72]-based their research on a 6-item measure proposed by Blau [12]. As a result, they received an aggregate measure that included answers to questions about completing an application and sending a CV to potential employers. Such an aggregate measure did not reflect how serious these intentions of leaving the job were. I suspect that affective commitment plays a mediating role in the initial stage of searching for a new job. An overqualified employee does not feel an emotional connection to the organization and starts thinking about changing jobs. His or her first move is to browse job listings in the press and on the Internet. Thus, I hypothesize the mechanism through which POQ affects browsing job offers:

### **H3**

The relationship between POQ and browsing job offers in the press and the Internet is fully mediated through organizational commitment.

**Fig. 2:**
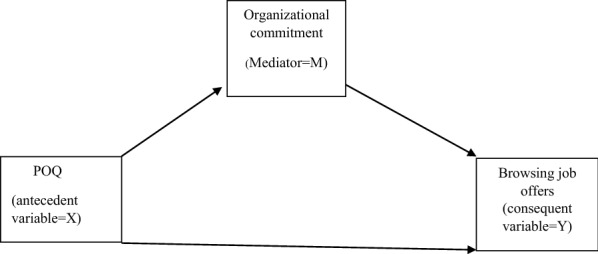
Conceptual model for the mechanism through which POQ affects browsing job offers in the press and the Internet (hypotheses 3)

Hypothesis 3 is illustrated in Fig. 2.

If organizational commitment proves to be a significant mediator, then the strengthening of the emotional connection between overqualified employees and the organization could help prevent such employees from seeking new job offers.

With regard to advanced job search, which is submitting an application for a new job, I suspect that organizational commitment is not a mediator. This is because if an overqualified employee eventually decides to apply for a new job, the main motivation for such an action is the belief his or her educational and professional experiences are not fully utilized. I therefore believe that the effect of POQ on applying for a new job is direct. It will be interesting to see if this direct effect can be weakened by improving the relations between superiors and employees or salary satisfaction. Thus, I hypothesize the mechanism through which POQ affects applying for a new job:

### **H4**

The direct relationship between POQ and applying for a new job is moderated by interpersonal justice.

### **H5**

The direct relationship between POQ and applying for a new job is moderated by pay satisfaction.

**Fig. 3:**
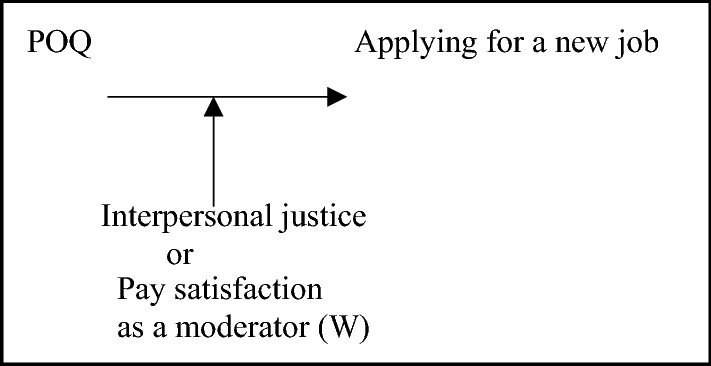
Conceptual model for the direct effect of POQ on applying a new job moderated by interpersonal justice (or pay satisfaction)

Figure 3 illustrates both hypotheses.

In the second part of this study, I look for answers to questions about how to strengthen the affective commitment through non-salary job attributes and pay satisfaction. If the relationships between perceived overqualification and turnover intention as well as browsing job offers are entirely mediated through organizational commitment, it can be assumed that it is possible to weaken the impact of POQ on turnover intention by strengthening the organizational commitment of overqualified employees. To this end, one can use non-salary job attributes (such as nonwage benefits and the elasticity of work hours) and pay satisfaction as well as improve fairness at the workplace. However, these job attributes can help to reduce retention only if they transmit the effect of perceived overqualification on affective commitment, i.e., if they are significant mediators. The results presented in the literature suggest that this idea can be validated in the current study.

Based on the model of effort-reward imbalance developed by medical sociologist Siegrist [97], I suspect that salary and nonwage benefits, such as healthcare package, sports tickets, etc., could reduce this type of imbalance experienced by overqualified employees. A mismatch between high effort spent at work and low gain received in turn makes overqualified employees feel negative emotions of reward frustration, which in turn causes job stress, which leads to a decrease in affective commitment. The negative effect of job stress on affective commitment was confirmed, for instance, by McLean and Andrew [75], Kaur [51], Yukongdi and Shrestha [110]. Job stress is one of the major organizational factors that increase turnover intention [6, 79, 40, 46,7].

The idea that elasticity of work hours could be used to reinforce affective commitment is based on the conservation of resources theory, developed by Hobfoll [42]. This theory states that people experience stress when they can't maintain or acquire resources that are important to them. Liu and Xi [64] showed that overqualified employees will feel stress at work because they cannot fully utilize two resources that are important to them, knowledge and time. If offered elasticity of work hours they would have more control over their work and consequently strengthen their affective commitment and weaken turnover intention.

Another potential mediator of the effect of perceived overqualification on affective commitment could be clear rules for promotion or organizational justice. Organizational justice theory indicates that overqualified personnel is inclined to perceive their working conditions as unfair [1]. Thus, reinforced procedural justice appropriateness in decision-making procedures, including promotion rules, [59, 100] could stop them from leaving their current job. This idea is supported by the results presented in the literature. Iyigun and Tamer [45] as well as Phayoonpu and Mat [85] showed the negative and statistically significant association between procedural justice and turnover intention. Also, in research conducted by Kwai et al. [57] procedural justice proved to be an important factor that increased organizational commitment and decreased turnover intention. The importance of procedural justice, primarily concerned with rules of promotion, is indirectly demonstrated by research results obtained by Maynard and Parfyonova [72]. They show that competence and growth value was a significant moderator for the impact of perceived overqualification on affective commitment and active job search behavior. Clear promotion procedures are important for career advancement, as research into the nature of the relationship between overqualification and career success shows that this relationship is negative [28, 36, 105]. If overqualified employees are convinced that promotion in their organization depends on appropriate qualifications, they will see opportunities for career advancement and feel more committed to the organization and consequently will be more likely to continue working there.

To answer the question whether non-salary work attributes could be used to strengthen organizational commitment and, consequently, to prevent overqualified employees from leaving their jobs, the hypotheses regarding the mediators of the relationship between POQ and organizational commitment will be verified (hypotheses 6–8). Additionally, the hypothesis about pay satisfaction as a mediator will also be tested (H9).

### **H6**

The relationship between perceived overqualification and organizational commitment is entirely mediated through nonwage benefits.

### **H7**

The relationship between perceived overqualification and organizational commitment is entirely mediated through the elasticity of work hours.

### **H8**

The relationship between perceived overqualification and organizational commitment is entirely mediated through procedural justice.

### **H9**

The relationship between perceived overqualification and organizational commitment is entirely mediated through pay satisfaction.

**Fig. 4:**
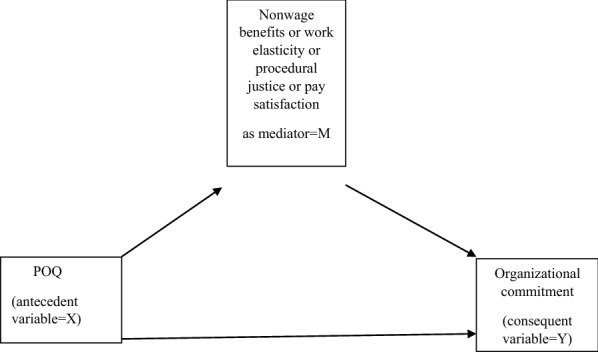
Conceptual model for the mechanism through which POQ affects organizational commitment (hypotheses 6–9)

A conceptual mediation model for the relationship between *perceived overqualification* and organizational commitment is presented in Fig. 4.

Finally, in the third stage of this study, if negative affectivity turns out not to be a significant moderator of POQ effects, then the results regarding the mechanisms through which POQ influences turnover intentions and job search behaviors will be considered robust. Thus, I hypothesize:

### **H10**

Negative affectivity (pessimism) is not a relevant moderator in the POQ-Turnover intention relationship and the POQ-Organizational commitment relationship.

A positive validation of *H10* would mean that the relationships between *POQ* and *Turnover intention* and between *POQ* and *Organizational commitment* are not spurious, i.e., they are not due to the fact that the variables in these relationships are correlated with the "third" variable, which is *Negative affectivity*. *H10* validation is a test that checks the robustness of the results obtained from the mediation models.

## Materials and method

### Sample

The data in this paper comes from a survey conducted using the CAWI (computer-assisted interview) method among people aged 25–45 (i.e., people of mobile working age) with higher education. The survey was conducted in Poland in November 2016 on a sample of 826 participants. A conscious selection of participants was based on random-quota and covered the whole country.

Overqualified employees have been identified from among all participants of the study on the basis of how much they agreed or disagreed with the following statements: (1) Current job suits you because of the use of your skills and experience; (2) Current job suits you because of the use of your knowledge and education. The participants used a five-point scale, where 1 = strongly disagree … 5 = strongly agree. Those participants who answered 1 = strongly disagree and 2 = disagree were considered overqualified employees. The scale was reversed (1 = disagree; 2 = strongly disagree) so that the increase in the overqualification measure matches the increase in the participant’s feeling that his or her skills and experience (or knowledge and education) are unused. The number of overqualified participants is 100, with respect to skills and experience, and 90, with respect to knowledge and education. The ratio of the overqualified in the total number of participants with higher education is 12%, considering skills and experience and 11%, considering knowledge and education.

The problem of overqualification affects women more than men. In a sample of 100 participants, women accounted for 63% while men accounted for only 37% (while in the baseline sample of 826 participants, there were 59% of women and 41% of men). In terms of age, twice as many young participants (aged 25–31) perceived themselves as overqualified (48%) compared to older groups, i.e., 24% of participants aged 32–38 and 28% aged 39–45. Only in the capital city the percentage of overqualified participants clearly drops (only 5%), while outside the capital there is little difference, i.e., 33% of overqualified participants live in large cities, 22% in medium-sized cities, 23% in small towns and 17% in rural areas. There is a clear disproportion in the distribution of overqualified participants in the employment sector. As many as 74% of overqualified are employed in the private sector, only 4% in the public sector, and 17% in state-owned enterprises. There is no such clear differentiation with regard to the size of the enterprise. In fact, clearly less, because only 10% of the respondents work in enterprises employing 500 or more people but the percentage of overqualified participants working in very small (up to 10 employees) and small enterprises (11–50 people) is the same (33%) and is lower (23%) only in medium-sized enterprises (51–500 people).

### The method

The research applied conditional process analysis, developed by Hayes [41], to test the research hypotheses on the relationships between perceived overqualification and turnover intention, perceived overqualification and organizational commitment, and between POQ and job search behaviors. The same method was used to test the hypothesis on the role of negative affectivity.

This method also includes moderation analysis which is used to verify the hypotheses regarding moderators in the POQ transmission mechanism on organizational commitment (see the conceptual model in Fig. 1).

It is also possible to investigate the moderation effect in the direct relationship between X and Y (see the conceptual model in Fig. 4).

### Definitions and measures of variables

Items in the survey were used to construct variable measures. A description of variable measurements is included in Table 1

**Table 1:** Measures of variables

Antecedent variable X (measured on 2-point scale: 1 = disagree; 2 = strongly disagree)	Item
POQ_SE_ (Perceived overqualification because of unused skills and experience)	How much you disagree with the statement: Current job suits you because of the use of your skills and experience
POQ_KE_ (Perceived overqualification because of unused knowledge and education)	How much you disagree with the statement: Current job suits you because of the use of your knowledge and education
Consequent variable Y	
	How much you disagree or agree with the statement:
Turnover intention	If the current job does not meet your expectations, then you are trying to find a job more suited to your expectations (1 = strongly disagree, …, 5 = strongly agree)
Browsing job offers in the press and the Internet Adapted from Blau [12] and Saks and Ashforth [94]	How often during the last 6 months did you browse job advertisements in the press or the Internet? 1 = never 2 = once 3 = several times 4 = many times
Applying for a new job Adapted from Blau [12] and Saks and Ashforth [94]	How often have you applied for a job in the last 6 months? 1 = never 2 = once 3 = several times 4 = many times
Mediators (measured on 5-point scale: 1 = strongly disagree, ……, 5 = strongly agree)	
Organizational commitment Adapted from [4] (!) Organizational commitment is a mediator in the relationships POQ_SE_/POQ_KE_ → Organizational commitment → Turnover intention/Browsing job offers/ Applying for a new job; see Table 3 and 4, while it is a consequent variable in the relationships POQ_SE_/POQ_KE_ → Mediator → Organizational commitment, see Table 6	How much you disagree or agree with the statement: I would like to spend the rest of my professional life in the company I am currently working in
Nonwage benefits	How much you disagree or agree with the statement: The organization you work for now provides you with additional benefits at an appropriate level, such as: healthcare package, sports tickets, etc
Elasticity of work hours	How much you disagree or agree with the statement: You have the opportunity to work at home if your personal situation does not allow you to come to work in the organization's seat
Procedural justice Based on Colquitt and Zipay (2015)	How much you disagree or agree with the statement: In the company where I am currently working, employees with the appropriate qualifications will be promoted
Pay satisfaction (!) Pay satisfaction is a mediator in the relationship *POQ*_*SE*_*/POQ*_*KE*_ → *Pay satisfaction* → *Organizational commitment* (see Table 6), while it is a moderator in the effects of *POQ*_*SE*_*/POQ*_*KE*_ on *Turnover intention/Applying for a new job*, see Tables 3 and 5	How much you disagree or agree with the statement: Current job suits you because of your income
Moderators (measured on 5-point scale: 1 = strongly disagree, ……, 5 = strongly agree)	
Interpersonal justice (respect from supervisors) Based on Colquitt and Zipay (2015)	How much you disagree or agree with the statement: Supervisors treat you with the same respect as others
Pessimism	How much you disagree or agree with the statement: I think I'm pessimistic
Control variable	
Gender	1 = Female 2 = Male
Age	25–45

The table above presents how the variables used in mediation models and moderation models are measured

## Results

### Organizational commitment as a mediator in the relationship *POQ–Turnover intention*

The results from this research confirm that relationship between perceived overqualification and turnover intention is entirely mediated through organizational commitment, considering both perceived overqualification because of unused skills and experience, POQ_SE_, as well as perceived overqualification because of unused knowledge and education, POQ_KE_ (see Table 2, both the indirect effect of POQ_SE_ = 0.2182 and the indirect effect of POQ_KE_ = 0.3836 are statistically significant, while both the direct effects are not). Therefore, hypothesis 1: *The relationship between perceived overqualification and turnover intention is entirely mediated through organizational commitment*, is confirmed.

**Table 2:** Direct and indirect effects of perceived overqualification on turnover intention with organizational commitment acting as a mediator

Antecedent variable = X	Consequent variable = Y = Turnover intention	
	Mediator = M = Organizational commitment X → Organizational commitment → Y	
	Direct effect	Indirect effect
POQ_SE_ (Perceived overqualification because of unused skills and experience)	.3609 (− .2213; .9430)	**.2182** (.0234; .4662)
POQ_KE_ (Perceived overqualification because of unused knowledge and education)	.0747 (− .4741; .6236)	**.3836** (.1526; .6038)

Statistically significant effects are in bold

In parentheses values of LLCI (lower limit confidence interval) and ULCI (upper limit confidence interval) for direct effects while values of BootLLCI (bootstrap lower limit confidence interval) and BootULCI (bootstrap upper limit confidence interval) for indirect effects

The significance of parameters at the level of 0.95

The results in Table 2 come from Hayes PROCESS for SPSS and SAS, model 4 (see Fig. 5); age and gender are statistically insignificant controls

Number of participants: N = 100 for POQ_SE_ and N = 90 for POQ_NE_

Source: author’s estimation

A deeper analysis including two moderators, *Pay satisfaction* and *Interpersonal justice*, shows (see Table 3) that the strongest conditional effect of perceived overqualification on turnover intention occurs when the overqualified employees are definitely dissatisfied with their income or when, in their opinion, they are clearly not treated well by their superiors compared to other employees. The moderation impact of *Interpersonal justice* (respect from supervisors) is stronger that the moderation impact of *Pay satisfaction*, but only in the relationship *POQ*_*SE*_ –*Turnover intention* (both moderation effects are not statistically significant in the relationship *POQ*_*KE*_ –*Turnover intention*)—compare the indices of moderated mediation in Table 3.

**Table 3:**
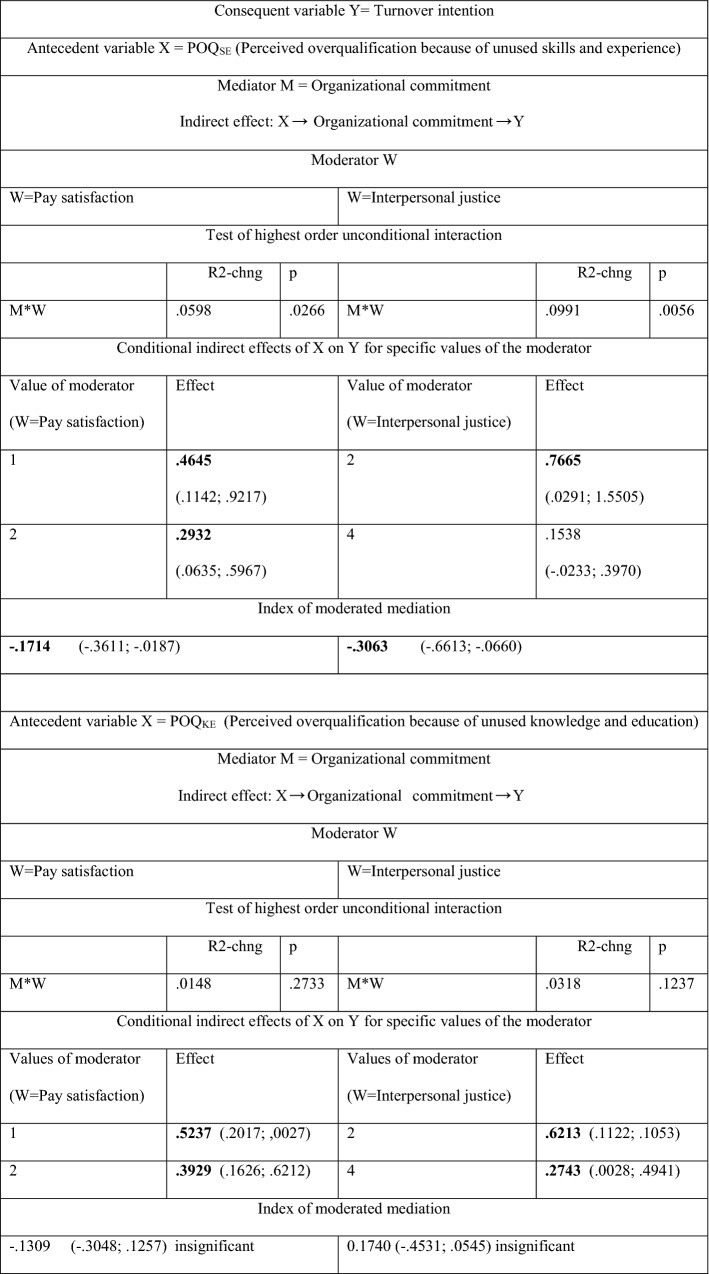
Conditional indirect effect of *Perceived overqualification* (because of unused skills and experience, *POQ*_*SE*_, and because of unused knowledge and education, *POQ*_*KE*_) on *Turnover intention*; moderators: *Pay satisfaction* and *Interpersonal justice* (respect from supervisors)

Statistically significant effects are in bold

Values in parentheses are BootLLCI and BootULCI

The significance of parameters at the level of 0.95

The results in Table 3 come from Hayes PROCESS for SPSS and SAS, model 14 (see Figs. 6 and 2); age and gender are statistically insignificant controls

See the definition of the index of moderated mediation in Hayes PROCESS for SPSS and SAS

Number of participants: N = 100 for POQ_SE_ and N = 90 for POQ_NE_

Source: author’s estimation

The importance of *Interpersonal justice* as a moderator is emphasized by test results (see Table 3), which show that, in most cases, overqualified employees (i.e., those who believe that their skills and experience are not properly used), if treated with respect by their superiors, do not plan to leave their organization (see Table 4, the conditional indirect effect of *X* = *POQ*_*SE*_ on *Y* = *Turnover intention* at the value of moderator = 4, (*Interpersonal justice,* as the moderator) equals 0.1538 and it is statistically insignificant BootLLCI = − 0.0233, BootULCI = 0.3970)).

**Table 4:**
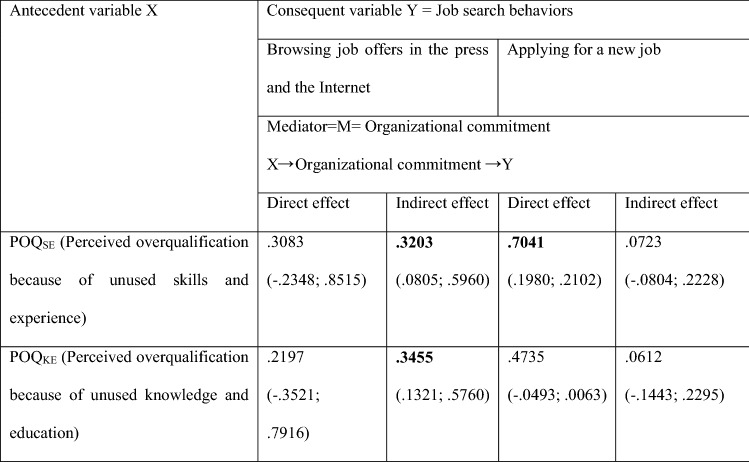
Direct and indirect effects of perceived overqualification on job searching behaviors with organizational commitment acting as a mediator

Statistically significant effects are in bold

The values in parentheses are LLCI and ULCI for direct effects and BootLLCI and BootULCI for indirect effects

The significance of parameters at the level of 0.95

The results in Table 5 come from Hayes PROCESS for SPSS and SAS, model 4 (see Fig. 5); age and gender are statistically insignificant controls

Number of participants: N = 100 for POQ_SE_ and N = 90 for POQ_NE_

Source: author’s estimation

### The mechanisms through which POQ influences job search behaviors

If the overqualified declare a desire to find a job that suits them better, then a question arises as to how active they are in their search for a new job. Are they just looking through job offers in the press or the Internet, or are they applying for a new job? What role does organizational commitment play in these behaviors?

The results of mediation analysis (see Table 4) indicate that only the relationship *POQ- Browsing job offers in the press and the Internet* is fully mediated through *Organization commitment*, while *Organization commitment* is statistically insignificant, as a mediator, in the relationship *POQ-Applying for a new job*. It is noteworthy that POQ_SE_ directly prompts the overqualified to apply for a new job, while POQ_KE_ has neither a direct or indirect effect (see Table 4).

A deeper dive into the direct effect of *POQ*_*SE*_ on *Applying for a new job* shows the importance of two moderators: *Pay satisfaction* and *Interpersonal justice* (see Table 5). A clear lack of satisfaction with the income obtained and a deficit of interpersonal justice strengthen the direct effect of *POQ*_*SE*_ on *Applying for a new job*. It is more likely for employees who believe that their skills and experience are not properly used to apply for another job if they are clearly dissatisfied with their pay and when they think they are treated with less respect than others.

**Table 5:**
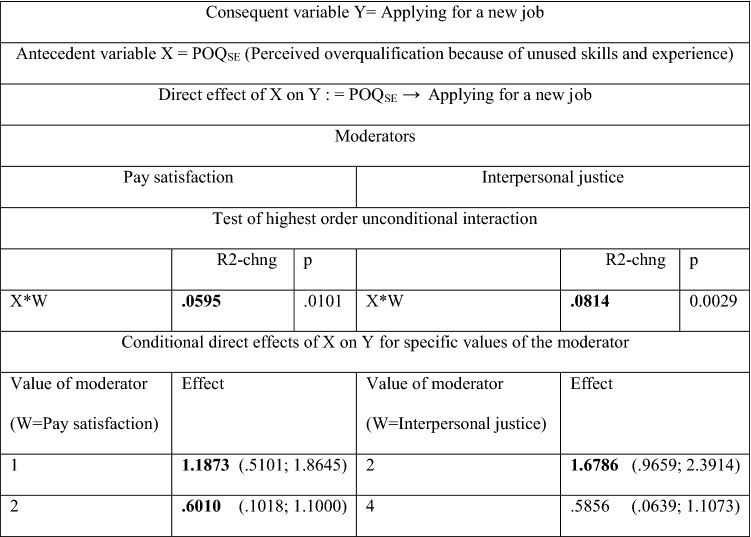
Moderated direct effect of *POQ*_*SE*_ (Perceived overqualification because of unused skills and experience) on *Applying for a new job* with the following moderators: *Pay satisfaction* and *Interpersonal justice* (respect from supervisors)

Statistically significant effects are in bold

Values in parentheses are BootLLCI and BootULCI

The significance of parameters at the level of 0.95

The results in Table 6 come from Hayes PROCESS for SPSS and SAS, model 1 (see Fig. 7); age and gender are statistically insignificant controls

Number of participants: N = 100 for POQ_SE_

Source: author’s estimation

### The mediation effects of non-salary and salary job attributes in the relationship *POQ—Organizational commitment*

The findings (Table 2) show that the relationship between perceived overqualification and turnover intention is entirely mediated through organizational commitment. Therefore, it can be assumed that it is possible to weaken the impact of POQ on turnover intention by strengthening the organizational commitment of the overqualified. In hypotheses 6–9, I test the mechanisms through which *POQ*_*SE*_ or *POQ*_*KE*_ influence *Organizational commitment*. The findings are presented in Table 6. They show that the relationship *POQ*_*SE*_* – Organizational commitment* is fully mediated through non-salary job attributes such as *Nonwage benefits*, *Elasticity of work hours* and *Procedural justice*. The strongest mediator is *Nonwage benefits* followed by *Procedural justice* and *Elasticity of work hours* (the indirect effects are − 0.6339; − 0.4147; − 0.3812, respectively, see Table 4). All three non-salary job attributes can be used as tools to increase organizational commitment; however, mainly for employees who believe that their skills and experience are not properly used. For employees who think their knowledge and education is not properly used, non-salary job attributes only partially transmit the impact of *POQ*_*KE*_ on *Organizational commitment*, or are irrelevant as a mediator. Therefore, these three job attributes will be much less effective in strengthening organizational commitment of these overqualified employees.

**Table 6:**
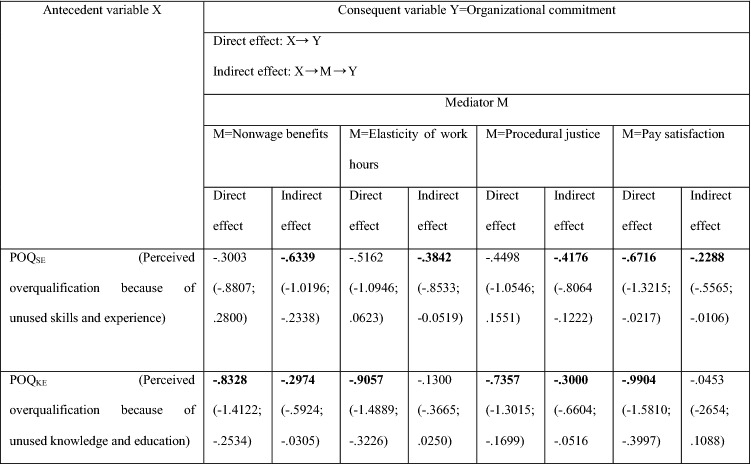
Direct effects of *Overqualification* ( POQ_SE_ or *POQ*_*KE*_) on *Organizational commitment* as well as indirect effects by one of four following mediators: *Nonwage benefits; Elasticity of work hours; Procedural justice; Pay satisfaction*

Statistically significant effects are in bold

Values in parenthesis are LLCI and ULCI for direct effects and BootLLCI and BootULCI for indirect effects

The significance of parameters at the level of 0.95

The results in Table 4 come from Hayes PROCESS for SPSS and SAS, model 4 (see Fig. 5); age and gender are statistically insignificant controls

Number of participants: N = 100 for POQ_SE_ and N = 90 for POQ_NE_

Source: author’s estimation

Considering the salary job attribute, *Pay Satisfaction* turned out to be a much weaker mediator (or even an insignificant mediator) in the relationship *POQ-Organizational commitment* (see Table 6). Thus, the use of non-salary job attributes seems to be a better instrument than the salary job attribute in increasing the organizational commitment perceived by overqualified employees, especially by those who feel that their skills and experience are not being properly used by their organization.

### Impact of pessimism on the relationships of *POQ–Turnover intention*

Due to their negative attitude toward many areas of their life, pessimists may believe that in the organization in which they work, they do not fully use their skills and experience as well as their knowledge and education. Such an assessment leads pessimists to low organizational commitment and is a strong incentive for them to leave the organization.

Therefore, pessimism would be the variable correlated with both POQ and organizational commitment as well as with turnover intention. Consequently, the POQ effects analyzed above would be spurious. To check if the results are robust, I started with a correlation and then used moderation analysis, where pessimism was the moderator.

First of all, the correlations between *Pessimism* and *POQ*_*SE*_*/POQ*_*KE*_ are statistically insignificant (− 0.064; − 0.030, respectively). *Pessimism* is not correlated with *POQ*. The moderation analysis shows that *Pessimism* is not also a significant moderator in the direct effect *of POQ* and *Organizational commitment* (see Table 7) as well as in the indirect effects of *POQ* on *Organizational commitment* through non-salary and salary job attributes (see Table 8).

**Table 7:** Moderated direct effect of POQ_SE_ (Perceived overqualification because of unused skills and experience)/ POQ_KE_ (Perceived overqualification because of unused knowledge and education) on *Organizational commitment*—*Pessimism* as a moderator

Antecedent variable X	Consequent variable Y = Organizational commitment		
	Direct effect: X → Y		
	Moderator W = Pessimism		
	Test of highest order unconditional interaction		
		R2-chng	p
POQ_SE_ (Perceived overqualification because of unused skills and experience)	X*W	.0080	.3008
POQ_KE_ (Perceived overqualification because of unused knowledge and education)	X*W	.0111	.2622

The significance of parameters at the level of 0.95

The results in Table 7 come from Hayes PROCESS for SPSS and SAS, model 1 (see Fig. 7); age and gender are statistically insignificant controls

Number of participants: N = 100 for POQ_SE_

Source: author’s estimation

**Table 8:**
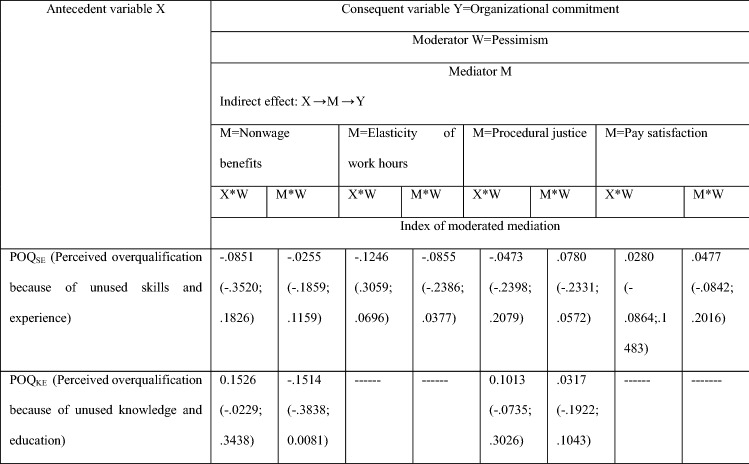
Moderated mediation effect of *Perceived overqualification* (because of unused skills and experience, *POQ*_*SE*_, and because of unused knowledge and education, *POQ*_*KE*_) on *Organizational commitment*; *Pessimism* as a moderator

Values in parentheses are BootLLCI and BootULCI. The significance of parameters at the level of 0.95

––- if the indirect effect X → M → Y is insignificant, see Table 5, therefore it does not make sense to test the moderated mediation effect

The findings in Table 8 come from Hayes PROCESS for SPSS and SAS, model 7 and model 14 (see Fig. 6); age and gender are statistically insignificant controls

Number of participants: N = 100 for POQ_SE_ and N = 90 for POQ_NE_

Source: author’s estimation

Moderation analysis (see Tables 9 and 10) reveals the difference in the role of pessimism as a moderator of the indirect *POQ* effect on *Turnover intention*, depending on whether employees think their skills and experience are not fully used (*POQ*_*SE*_), or that the job they do does not require the level of knowledge and education they have achieved (*POQ*_*KE*_). *Pessimism* does not moderate the indirect effects of *POQ*_*SE*_ on *Turnover intention* (see Table 9). However, when employees perceive themselves as overqualified due to underutilized knowledge and education (*POQ*_*KE*_), then pessimism is an important moderator in the causal chain of events *POQ*_*KE*_ → Organizational commitment → Turnover intention (see Table 10). Pessimism moderates this causal chain of events twice, once when it moderates the relationship *POQ*_*KE*_ → Organizational commitment (X*W), and the second time when it moderates the relationship *Organizational commitment* → *Turnover intention* (M*W).

**Table 9:** Moderated mediation effect of *POQ*_*SE*_ (*Perceived overqualification* because of unused skills and experience) on *Turnover intention*; *Pessimism* as a moderator

Antecedent variable X	Consequent variable Y = Turnover intention	
	Moderator W = Pessimism	
	Mediator M = Organizational commitment Indirect effect: X → M → Y	
	X*W	M*W
	Index of moderated mediation	
POQ_SE_ (Perceived overqualification because of unused skills and experience)	− .0540 (− .0522; .1659)	− .0609 (− .2527; .0952)

Values in parentheses are BootLLCI and BootULCI. The significance of parameters at the level of 0.95

The results in Table 9 come from Hayes PROCESS for SPSS and SAS, model 7 and model 14 (see Fig. 6); age and gender are statistically insignificant controls

Number of participants: N = 100 for POQ_SE_ and N = 90 for POQ_NE_

Source: author’s estimation

**Table 10:**
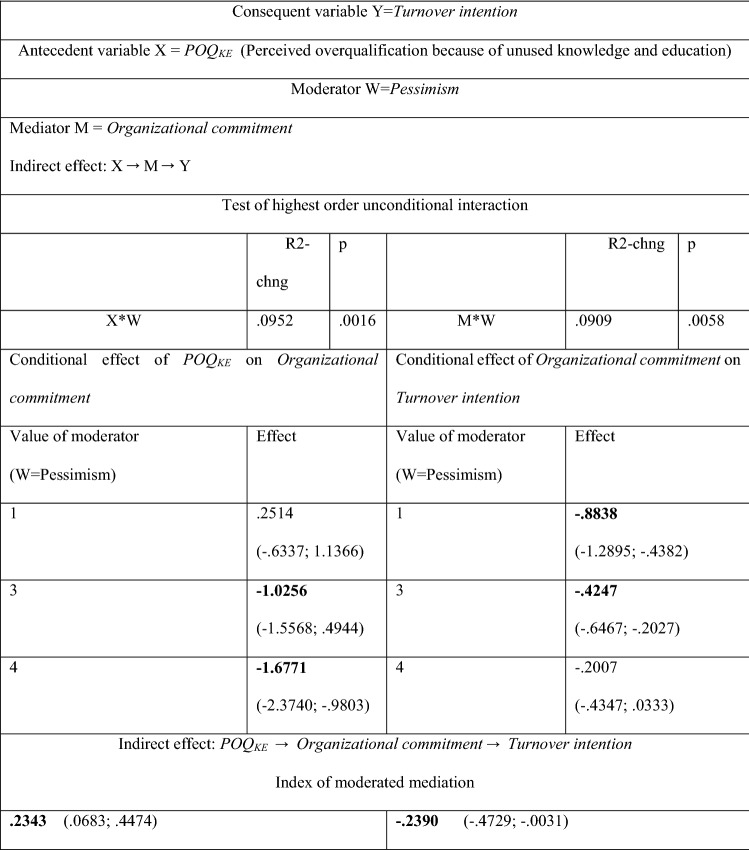
Moderated mediation effect of *POQ*_*KE*_* (Perceived overqualification* because of unused knowledge and education, *POQ*_*KE*_) on *Turnover intention*; *Pessimism* as a moderator

Values in parentheses are BootLLCI and BootULCI. The significance of parameters at the level of 0.95

The results in Table 10 come from Hayes PROCESS for SPSS and SAS, model 7 and model 14 (see Fig. 2); age and gender are statistically insignificant controls

See the definition of the index of moderated mediation in Hayes PROCESS for SPSS and SAS

Number of participants: N = 100 for POQ_SE_ and N = 90 for POQ_NE_

Source: author’s estimation

Interestingly, the high level of *Pessimism*, on the one hand, intensifies the negative impact of *POQ*_*KE*_ on *Organizational commitment*, while on the other hand, it reduces the impact of *Organizational commitment* on *Turnover intention*. The strength of these two moderation effects is very similar (the indices of moderated mediation are 0.2343 and − 0.2390, respectively, see Table 10). Consequently, these effects are almost in balance and it can be assumed that ultimately *Pessimism* also does not moderate the indirect effect *POQ*_*KE*_ on *Organizational commitment*.

It is worth to analyze in depth the impact of *Pessimism* when it moderates the causal chain of events *POQ*_*KE*_ → *Organizational commitment* → *Turnover intention (see* Table 10). While the perception of yourself as an employee who is clearly overqualified, reduces the organizational commitment of pessimists (as expected), pessimists do not declare a strong turnover intention (which is unexpected). In Table 10, the conditional impact of *POQ*_*KE*_ on organizational commitment clearly increases with the increase in the value of the *Pessimism* variable. When the employee considers him or herself a pessimist (value of the variable *Pessimism* = 4), the negative influence of *POQ*_*KE*_ on *Organizational commitment* is the strongest (effect = − 1.6771). Interestingly, overqualified pessimists, have in fact a low organizational commitment but do not show willingness to leave the job—see Table 10 where the conditional effect of *Organizational commitment* on *Turnover intention* (− 0.2007) is irrelevant for the value of the variable *Pessimism* = 4. Conversely, if overqualified personnel perceives themselves as optimists (value of *Pessimism* = 1), then their low rating of organizational commitment prompts them to give up their jobs in their ex organization. (The conditional effect of *Organizational commitment* on *Turnover intention* = − 0.8838). This conclusion is confirmed by the moderated direct effect of *POQ*_*SE*_ (Perceived overqualification because of unused skills and experience) on *Applying for a new job*. This effect is the highest (1.2733) for the value of the variable *Pessimism* = 1, but it is irrelevant when the variable *Pessimism* = 4—see Table 11. Optimists, not pessimists, more often decide to find another job if they are overqualified.

**Table 11:**
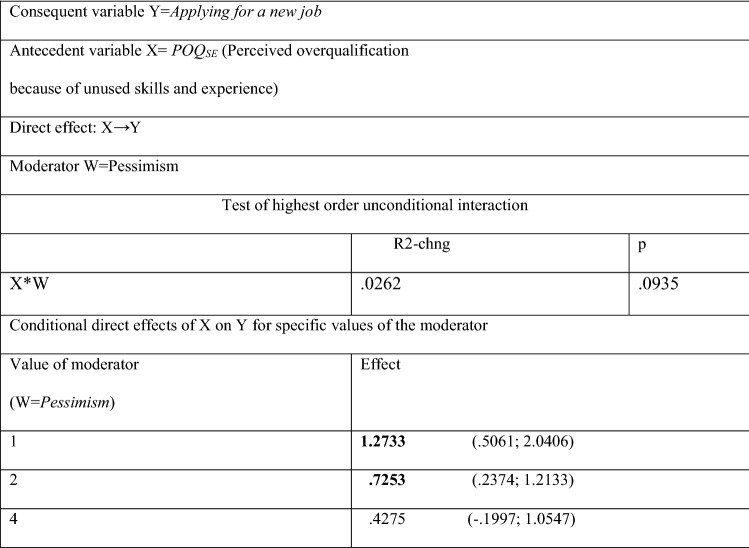
Moderated direct effect of *POQ*_*SE*_ (Perceived overqualification because of unused skills and experience) on *Applying for a new job*—*Pessimism* as a moderator

The significance of the parameters at the level of 0.95

The results in Table 11 come from Hayes PROCESS for SPSS and SAS, model 1 (see Fig. 3); age and gender are statistically insignificant controls

Number of participants: N = 100 for POQ_SE_

Source: author’s estimation

Summing up the results, it can be concluded that the impact of pessimism on the studied *POQ* relationships does not occur and therefore these relationships can be considered robust. In their meta-analysis, Harari et al. [39, p. 43] also found that the relationship between POQ and turnover intention as well as POQ and organizational commitment does not result from the effect of negative affectivity on the variables in those relationships.

### The relevance of gender and age

Although in the surveyed sample of overqualified employees, the share of women was much higher than that of men (63% and 37%, respectively), gender, as a control variable, was statistically insignificant in all mediation models. This is consistent with the results of the meta-analysis by Harari et al. [39].

Age was also an insignificant control variable although 45% of the overqualified participants were aged between 25 and 31.

The findings of this paper suggest that employees who have attained higher education, regardless of gender and age, respond in a similar manner when overqualification becomes their problem.

## Discussion

In the discussion of the obtained results, I concentrate on three issues. First, I try to find an explanation for the difference between the effects produced by overqualification caused by unused knowledge and education and the effects produced by overqualification resulting from unused skills and experience. Second, I try to answer the question of whether an organization should retain overqualified employees. Third, I approach the problem of overqualification from the point of view of a professional career and the work/life balance.

I support my explanation with a qualitative characterization of the participants of this research included in Table 12. In it, I present the responses to items obtained as a result of a questionnaire survey in which overqualified employees participated. I focus on definite responses to items stating only the percentage of participants, who responded with *disagree* + *strongly disagree* and *agree* + *strongly agree*.

**Table 12:** Qualitative characterization of the participants in the current study

Item	Perceived overqualification because of unused skills and experience, *POQ*_*SE*_		Perceived overqualification because of unused knowledge and education, *POQ*_*KE*_	
	How much you disagree or agree with the statement:			
	Percentage of participants who answered			
	disagree + strongly disagree	agree + strongly agree	disagree + strongly disagree	agree + strongly agree
**Opportunities for professional development**				
In my current workplace, I have ample opportunity for professional development	57%	23%	66%	20%
I will have the opportunity for promotion within the next year	59%	23%	62%	18%
My current job meets my expectations and needs regarding opportunities for advancement and career development (*adapted from Saks and Ashforth*, [94])	40%	38%	52%	27%
Employability: I have the opportunity to find a new and better job	27%	47%	32%	47%
In the future, including other situations than my current job, I will have great opportunities for professional development and career plan implementation	35%	38%	36%	37%
My employer offers a number of good training courses that I can participate in to improve my skills	48%	29%	62%	20%
**The beginning of a professional career**				
Higher education was required of applicants for your first ever job that you got after completing your education	74%	26%	81%	19%
Higher education was actually needed for your first ever job that you got after completing your education	65%	35%	79%	21%
**Career plan**				
I have a plan for developing my career	31%	44%	38%	41%
** Deprivation** (*higher percentage of disagree* + *strongly disagree responses means higher deprivation*)				
Deprivation due to employment conditions: My employment conditions (tasks, responsibilities, promotion opportunities, salaries, bonuses) are similar to those of people with the same qualifications in other organizations	39%	23%	42%	20%
Deprivation due to financial well-being: I am wealthier compared to the general population of my local area	46%	15%	51%	14%
**Distributive justice**				
Considering the workload of the assigned tasks, my colleagues receive higher salaries and/or bonuses	37%	29%	39%	26%
My co-workers have greater opportunities to use their skills	33%	38%	37%	34%
**Emotional outcomes of perceived overqualification**				
Depression: I feel depressed when I have to do unimportant work (*adapted from Entrepreneurial Attitude Orientation Scale, developed by *Robinson et al. [91])	43%	51%	32%	43%
Life satisfaction: My life so far has been good	7%	65%	10%	62%
Financial satisfaction: I am satisfied with my financial situation	41%	37%	48%	31%
Burnout: In recent months, I have felt an excess of responsibilities at work that I could not cope with	68%	0%	68%	0%
**Positive behavioral responses to perceived overqualification**				
Job crafting: You are trying to adjust the work you do so that it gives you more satisfaction	17%	38%	22%	31%
You do extra work to get a promotion (or get a full-time position) as soon as possible	26%	28%	30%	22%
**Negative behavioral responses to perceived overqualification**				
You are dissatisfied and perform the assigned tasks with as little effort as possible	25%	34%	28%	39%
**Overall assessment of the job**				
I would describe my job as good	46%	23%	44%	24%
In my current job, I have the opportunity to do what I want and enjoy doing (*adapted from Saks and Ashforth*, [94])	40%	35%	46%	27%
**Relations with the team**				
I and the group of people I work with agree on the amount of work that needs to be put into the tasks we are assigned (*adapted from Saks and Ashforth*, [94])	23%	46%	23%	44%
I feel strongly connected to the people I work with *(based on *Brawley et al. [14]	34%	36%	37%	34%
The way my immediate supervisor manages is in line with my expectations (*adapted from Saks and Ashforth*, [94])	27%	53%	33%	33%
**Quality of education**				
The college/university where I studied offered a high quality of instruction and ranked high among universities in the country	25%	45%	28%	38%
Studies, training, postgraduate studies have improved my qualification	11%	67%	13%	60%
**Innovativeness** *(adapted from Entrepreneurial Attitude Orientation Scale, developed by *Robinson et al. [91]				
I get tired of working with people who have lots of new ideas	37%	36%	42%	28
I prefer to join a group people who are already carrying out a project rather than propose a completely new idea	26%	45%	24%	40%
The lack of challenges in my job affects my evaluation of the work I am currently performing	7%	26%	7%	28%
**Achievement** *(adapted from Entrepreneurial Attitude Orientation Scale, developed by *Robinson et al. [91]				
I believe that specific results are necessary to be able to evaluate whether something is successful or not	13%	67%	14%	69%
I often sacrifice my private time and personal convenience to take advantage of professional opportunities that come my way	30%	55%	29%	52%
**Personality**^(1)^ *Based on: Facets of personality by* Gosling et al. [35]. *Locus of Control Scale by *Rotter [92]*,Self-esteem measure by *Robins et al. [90]*,Risk aversion measure by *Greco and Roger [37]*,Procrastination items adapted from *Tuckman, [101]				
Antagonism: I consider myself critical of others, confrontational	29%	19%	34%	13%
Conscientiousness: I consider myself a conscientious, disciplined person	7%	41%	7%	39%
Openness: I consider myself open to new experiences, with a complex view of the world	10%	46%	9%	42%
Agreeableness: I consider myself a conciliatory, kind-hearted person	6%	47%	4%	51%
External locus of control: I often feel that I have little influence over what happens to me Sometimes I feel that I don't have enough control over the direction my life is taking	21% 21%	58% 53%	24% 26%	51% 49%
Self-esteem: I have high self-esteem	12%	75%	12%	68%
Risk aversion: When making a decision, I am deterred by the fear of making mistake	18%	58%	22%	49%
Procrastination: When I make an action plan, I try to follow it It's not in my nature to procrastinate	8% 16%	74% 55%	8% 21%	69% 52%
**Values of life** *(adapted from *[10]				
In life, I value financial comfort that allows me to possess things, which I really care about	16%	64%	16%	60%
I value owning property in life	18%	56%	23%	54%
I value a career in life (achieving success in professional work)	14%	62%	16%	62%
I value respect and appreciation from other people in life	12%	66%	10%	64%
I value friendship in life	14%	71%	9%	71%
I value family life	8%	74%	10%	71%
**Work-Welfare Indicators** (*by Baron, 2008)*				
Parental education is very important	26%	51%	34%	43%
Own education is very important	16%	72%	13%	68%
Own ambition is very important	14%	67%	13%	66%
Having a job is very important	10%	79%	10%	80%
Having a wealthy family is very important	23%	63%	24%	61%
**Conflicts in the family**				
Financial matters often cause quarrels in your family	50%	28%	53%	28%

Source: Author’s calculation based on the survey conducted using the CAWI (computer-assisted interview) method among people aged 25–45 (i.e., people of mobile working age) with higher education in Poland in 2016

N = 100 Number of participants who felt overqualified because of unused skills and experience

N = 90 Number of participants who felt overqualified because of unused knowledge and education

^(1)^ The purpose of the current study is not to accurately measure the personality characteristics of the study participants therefore only selected items concerning locus of control, risk aversion and procrastination are included

With regard to the first issue, the obtained results indicate that the use of non-salary job attributes, as tools to prevent overqualified employees from leaving the organization, would be more effective with regard to employees who feel that their skills and experiences are unused. In contrast, the effectiveness of these tools would be lower for employees who feel that their knowledge and education are unused. This raises the question of why it is more difficult to incentivize employees with unused knowledge and education to want to stay with the organization, that is, to make them show stronger affective commitment, than employees who feel overqualified because of unused skills and experience? A qualitative characterization of the participants (see Table 12) indicates that this may be due to a difference in how they perceive professional development opportunities. Such an opportunity is not available in their organization according to a larger percentage (66%) of employees who believe that their knowledge and education are unused. Similarly, such prospects for development are not available according to employees dissatisfied with the use of their skills and experience, but their percentage is smaller (57%). Similarly, a higher percentage (52%) of those dissatisfied with the use of their education and skills feel that their organization does not meet their expectations in terms of opportunities for advancement and career development, while 40% of those dissatisfied with the use of their skills and experience are of a similar opinion. There is also a difference when evaluating the quality of training offered by the organization. The opinion that they do not improve skills is shared by a higher percentage (62%) of those dissatisfied with the use of their knowledge and education, while such a negative assessment is expressed by fewer (48%) of those dissatisfied with the use of their skills and experience. The above characterization of employees with unused knowledge and education suggests that they will be less inclined to be committed to an organization that does not offer them opportunities for advancement or improve their skills. They probably do not have the best experience related to affective commitment from their first job. The vast majority of them (80%) worked in positions where higher education was not required. In their current job, 42% feel deprivation due to conditions at work. Half of them experience a sense of deprivation due to material circumstances (51%) and feel financial dissatisfaction (48%). Feelings of deprivation and financial dissatisfaction are somewhat lower in the case of employees with unused skills and experience. The failure to use their knowledge and education in the current job may be the reason for a lower evaluation of the quality of obtained education. Employees with unused skills and experience tend to better assess their university, training, and postgraduate studies.

The practical value of the results obtained in this study depends on the answer to the following question: from the organization's perspective, are overqualified employees a liability or an asset for the organization? Overqualification can generate both negative and positive effects. According to the liability view, overqualification leads to low quality individual outcomes, which in turn lowers the outcomes of the entire organization. There are studies in literature that support this point of view. In their meta-analysis, Harrari et al. (2017) found a meta-analytic correlation of 0.16 between overqualification and counterproductive work behaviors, that were damaging to an organization (e.g., absenteeism). Erdogan et al. [25] cites several studies, in which researchers found a positive relationship between overqualification and counterproductive work behaviors [49, 62, 66]. Cheng et al. [20] indicates that overqualified employees high in need for achievement show a propensity for cyberloafing (wasting time at work by surfing the internet, shopping online), which decreases organizational effectiveness. The results obtained by Fine and Edwards [34] undermine the above negative assessment as they indicate that overqualified employees commit minor types of counterproductive work behaviors, which are not illegal or more serious in nature, or cause harm to co-workers. Belfield [9] points out another negative effect associated with hiring overqualified employees. They are likely to leave the organization, so they generate a high recruitment cost. Finally, there is the key issue of how overqualification affects job performance, i.e., to what extent do overqualified employees perform their duties at work. If a motivation approach is used [108], then a concern arises that a sense of deprivation will demotivate overqualified employees and consequently reduce performance, but if a capability-based approach is used, then research indicates a positive relationship between overqualification and performance [24, 26, 32, 33, 44, 65]. A number of studies has results confirming that managers evaluate overqualified employees as high performers [33, 43]. In their synthesis of the literature, Erodogan and Bauer [25], summarizing research on the positive relationship between overqualification and performance, make a conclusion that is important with respect to the practical significance of the results of this study. The relationship between overqualification and performance can be positive under the right conditions. This study indicates the possibility to use non-salary job attributes to create just such right conditions.

The creation of a job environment in which employees are connected to the organization will make sense when overqualification is viewed from an investment/asset perspective, in which case overqualified employees are seen as capital that strengthens the organization. This is because they have superior skills and qualifications, which contribute to a high self-esteem. Erdogan et al. [29] found that high levels of person-organization fit weakens the negative effect of perceived overqualification on the desire to be helpful. In their discussion of the results of research on the effects of overqualification on organizational citizenship behaviors Erdogan and Bauer [25] demonstrate that individual identification with the team is a factor that incentivizes overqualified employees to share their knowledge and skills with z co-workers.

Ng and Feldman [80] see a positive aspect of overqualification in the personality of the potential employee. They point out that employers hire people with university degrees for jobs that do not require such high qualifications, because they assume that a university degree indicates desirable workplace traits such as conscientiousness.

If overqualified employees are to be assets for an organization, it would be good if they were proactive and innovative. Zhang et al. [111] showed that they exhibit coworker- and organization-directed proactive behaviors, because they are convinced that they could take part in projects involving tasks that go beyond those included in their job descriptions. Luksyte and Spitzmueller [67] underline that overqualified employees demonstrate higher levels of creativity if they receive support from the organization. Lin et al. [61] showed that they undertake task crafting, which is a manifestation of their creativity, the more readily they identify with their organization. Erdogan and Bauer [25] note that researchers dealing with the relationship between overqualification and creative behaviors, emphasize that employee personality and organizational commitment significantly affect the nature of this relationship.

Kulkarni et al. [50, 55, p. 5240] summarize the research that adopted the investment/asset perspective with the apt statement that “hiring overqualified individuals is like buying an option on knowledge and experience that is not fully utilized in the current environment but that can be exercised in the future as the organization’s environment and thus workforce needs change.”

Based on research presented in the overqualification literature, it seems adequate to try and answer the question of whether overqualified employees, who are participants in this study, are a liability or an asset for their organizations? To begin with, let us examine the arguments for them being a liability, which implies there is no point in trying to stop them from leaving their jobs. About 40% clearly experience deprivation due to conditions at work. About 30% feel that they face distributive injustice, i.e., given the workload required to complete the assigned tasks, their co-workers receive higher compensation and/or bonuses. Almost half are depressed by the necessity to complete unimportant tasks. Between 40 and 50% are not satisfied with their financial situation. A third of the respondents admit that they perform assigned tasks with as little commitment as possible. Only about 30% say they are connected to their team. Organizations should expect that a significant portion of employees with such characteristics will leave, especially considering the fact that about 40% of them performs work they do not like and almost half of the survey participants believe they have the opportunity to find a new, better job.

However, there are also arguments for viewing overqualified participants as assets. The vast majority exhibits desirable workplace traits. 75% are goal-oriented (achievement), about 70% say they have high self-esteem, 40% consider themselves disciplined and conscientious, while half consider themselves compliant and friendly. More than half do not think they are prone to procrastination. The vast majority believe that individual and family background is important to get ahead in life, i.e., what matters in life is one’s own education but also that of one's parents, ambition, having a job and a wealthy family. Do participants see themselves as innovative? They are not leaders who propose innovative solutions, about 40% say they prefer to join people who are already implementing a project rather than propose a completely new idea, but a third are willing to work with people who initiate such ideas. More than 40% view themselves as open to new experiences. Thus, if supported by their organization, such people could become involved in new projects. Especially since almost half say that when working in a team they agree on the amount of work that is required to complete the tasks that the team is assigned. Do participants take on "small" tasks that make their job more rewarding? More than 30% undertake job crafting and less than 30% take on additional tasks to get a promotion sooner or earn a full-time position. There is one more important consideration. Participants do not experience burnout at work and they do not have an excess of responsibilities that they could not handle.

The presented qualitative characterization of the study participants allows us to consider overqualified employees with higher education as an asset for the organization. Thus, it is important to know the tools that can help to retain such employees in the organization. The results obtained in the current study can serve as a valuable clue.

Regarding the third issue, it is important to consider how the problem of overqualification should be approached by those employees who are affected by it? After all, the perception of overqualification depends on the perspective they take. Will they evaluate their situation at work through the lens of career success or work/life balance. This decision will depend on their values in life. If they believe that a career is very important in life, they will treat working in a position below their qualifications as a missed opportunity for professional success, to which they are entitled. The negative relationship between perceived overqualification and perceived career success is confirmed by researchers who study the nature of this relationship [28, 36, 89, 105]. The lack of a significant relationship is only suggested by Wassermann et al. [106]. In the economics literature there is the hypothesis put forward by Sicherman and Galor [95], which suggests that overqualification would be short-lived phenomenon in the life of an employee and overqualification would allow such a person to quickly transfer to a position where he or she could make proper use of his or her skills, a but the proposition has not been confirmed by empirical research, as Erdogan and Bauer [25] stated in their synthesis of the overqualification literature.

Not all employees think that career is the most important thing in life. There are those who would like to achieve work-life harmony, or have a healthy work-life balance. They believe that they work in order to live and not the other way around. They want to combine the social working environment factor in work-life and the friendships factor in private life. They may place more emphasis on family than on career. A number of studies show the positive aspect of overqualification, if it is chosen freely. Maltarich et al. [69], 70 point out that “intentional mismatch” can occur. In such cases overqualified employees accept their situation because it allows them to obtain a desired fit between the employees’ non-work values and interests and their working conditions.

The participants of this survey include people who are career-oriented. About 60% value achieving career success, financial comfort and owning property in life. More than 40% say they have a career plan. Over 60% believe that ambition is important in life. About 60% of participants do not see the possibility of promotion within the next year. 40% feel that their organization does not meet their needs regarding skills improvement. Almost half see the possibility of finding a new and better job. Some of these people are likely to leave the organization in order not to waste their time, and this would be the right decision. Overqualification, in their case, leads directly to applying for a new job, as this study confirms. If the organization recognizes such people as assets, it can try to prevent them from looking for a new job by raising their salaries and requiring managers to treat such people with respect, especially since 60% of participants believe that respect and appreciation of others is important in life. Should overqualified employees who are career-oriented stay in the organization? I think that the answer is yes but only if there is a likelihood that Sicherman and Galor’s hypothesis is correct and such employees will quickly move to positions that are adequate for their qualifications.

It seems that a large number of the participants of this survey are focused on achieving a work-life balance. Despite their sense of being overqualified over 60% rate their lives as good. More than 70% value family life and friendship. Flexible working hours should be an effective tool to increase their organizational identification.

Among the survey participants, there is a group of people who accept their situation at work. For them overqualification is this “intentional mismatch.” About 30% say they do what they like at work. 25% give a positive answer to the general question whether they consider their work to be good. They are satisfied with interpersonal relations at work. More than 30% feel connected with the ream and do not feel that their co-workers have better opportunities to use their qualifications. About 30% accept the management style of their immediate supervisor. More than 30% can make their work more satisfying (job crafting). More than 30% feel financial satisfaction and half have no family quarrels due to finances.

In conclusion, there is no clear answer to the question of whether overqualified employees should leave the organization or continue working there. Those who are focused on a successful career should look for a job that is appropriate for their qualifications, while those who prefer a good work-life balance and are satisfied with their work environment may choose to stay with their organization.

## Research contribution

This research contributes to the overqualification literature as it sheds light on several unclear issues. Firstly, the POQ literature shows different results on the direct and indirect effects on turnover intention. These findings support the indirect effect of perceived overqualification on turnover intention through organizational commitment, considering both perceived overqualification because of unused skills and experience as well as unused knowledge and education. Secondly, the area of moderators in the relationship POQ-Turnover intentions has not been researched in depth. This article provides new insight into the importance of interpersonal justice as a moderator. Thirdly, the findings confirm that the relationships between perceived overqualification and the studied variables do not result from the shared influence of pessimism (negative affectivity).

This study also expands knowledge on POQ relationships. Based on the findings which show the significance of individual non-salary job attributes, I was able to identify nonwage benefits as a tool with the strongest impact on the organizational commitment of the overqualified. What was also revealed are the mechanisms through which POQ influences job search behaviors.

## Limitations and further studies

The sample of overqualified participants was chosen randomly, which is an advantage, but its small size (N = 100) can be considered a limitation. However, the argument that parametric tests are robust, even on small samples, is provided by Norman [81], who based his conclusion on an extensive review of the results of empirical research spanning nearly 80 years.

A more serious limitation is the reliance only on cross-sectional data. Consequently, the results show the POQ mechanism only at a given point in time. Another limitation is the selection of participants. It was the intention of the author to include only people with higher education, at an age when they can fully use their professional skills. The question to consider, therefore, is whether the results obtained in this study have the value of generality. This is because they are based on data collected in a single country, Poland, at a single point in time, November 2016. At that time, the labor market in Poland was relatively good for workers. The unemployment rate fell steadily throughout 2016 and at the end was around 8%. It can be assumed that in such a labor market, workers with higher education may have a chance to find jobs that match their qualifications. As Vaisey [103] has shown overqualification rates change with the unemployment rate. In a relatively good economic situation, it is likely that organizations will treat overqualified employees with higher education as their assets and will try to retain them. Thus, the knowledge of job attributes that can be used for this purpose is desirable. The results of the current study are therefore of practical value when the economy is good.

But can they serve as a recommendation for organizations during a crisis? To date only Chia-Huei Wu et al. [109] studied overqualification during the COVID-19 crisis based on two samples, from the UK and USA. In their study, they emphasize that in difficult periods such as the COVID-19 crisis, mitigating negative job attitudes and reluctance to undertake activities that are beyond established responsibilities is important for the survival of the organization. It is therefore important to strengthen in overqualified employees the level of felt obligation to the organization. To this end, they proposed, to use self-sacrificial leadership, i.e., supervisors should motivate employees to devote themselves for the good of the collective Choi and Yoon, [78]. Thus, in times of crisis, overqualified employees can also be an asset for an organization. Even if they are unable to find another job in difficult times, low affective commitment will prompt them to leave when the labor market improves. Losing employees can be an important factor that inhibits an organization's growth after a crisis. In Poland, for example, after the COVID-19 crisis the demand for workers increased sharply and the unemployment rate dropped to 5% in 2021 and was below 3% in June 2022. Many companies were experiencing a serious shortage of workers. Therefore, the recommendations regarding job attributes that could effectively strengthen the employee's identification with the organization are also relevant in times of crises.

Future research should focus on expanding knowledge on how to manage overqualified employees. It can include personality characteristics, such as innovativeness and achievement-orientation. There is a need for further studies on the moderators of the POQ-retention relationships.

## Recommendation for practice

Based on the results, it is possible to formulate some recommendations for an organization to make overqualified employees feel more connected to it. These recommendations are valid both when the economic situation is good and in times of crisis. The above discussion shows that overqualified employees should be treated as human capital that the organization can use when it needs to change. Thus:

- Care should be taken to ensure that nonwage benefits are more valued and better offset overqualification.
- Procedural justice must be ensured so that the overqualified see a clear relationship between qualifications and promotions and believe in the possibility of promotion.
- Proposing flexible working hours can reduce the negative impact of overqualification on turnover intentions.
- Non-salary job attributes can have a stronger impact on organizational commitment than an increase in satisfaction with salary.
- Particular attention should be directed to interpersonal justice so that an overqualified employee feels that he/she is treated with the same respect as others
- Optimistic overqualified employees tend to apply for new jobs more often than pessimists.

## Conclusions

Overqualification, in general, by lowering organizational commitment leads to declarations of turnover intention. However, only overqualification due to unused knowledge and education leads directly to an application for a new job, while overqualification due to unused skills and experience encourages people to only browse job offers.

The relationship between perceived overqualification and turnover intention is entirely mediated by organizational commitment. This creates the opportunity to raise organizational commitment through non-salary job attributes, such as nonwage benefits, elasticity of working hours, and procedural justice. Improving working conditions can discourage the overqualified employees from leaving the organization.

Negative affectivity (pessimism) does not create a common tendency in POQ and retention, which is why POQ effects on retention, revealed in this research, can be considered robust.

## Data Availability

The datasets used and/or analyzed during the current study are available from the corresponding author on reasonable request.

## Abbreviations

POQ: Perceived overqualification
NA: Negative affectivity
AOC: Affective organizational commitment
KSAOs: Knowledge, skills, abilities, and other characteristics of an employee
POQ_SE_: Perceived overqualification because of unused skills and experience
POQ_KE_: Perceived overqualification because of unused knowledge and education
LLCI: Lower limit confidence interval
ULCI: Upper limit confidence interval
BootLLCI: Bootstrap lower limit confidence interval
BootULCI: Bootstrap upper limit confidence interval
